# Visual coronary calcium scoring to support opportunistic CAD screening: comparative evaluation of three established systems and introduction of a novel scoring system

**DOI:** 10.1016/j.ijcha.2026.101875

**Published:** 2026-01-19

**Authors:** Philipp Nicol, Rafael Adolf, Salvatore Cassese, Adnan Kastrati, Michael Joner, Heribert Schunkert, Martin Hadamitzky, Leif-Christopher Engel

**Affiliations:** a Department of Cardiovascular Radiology and Nuclear Medicine, German Heart Center Munich, TUM University Hospital, Munich, Germany; b Department of Cardiovascular Diseases, German Heart Center Munich, TUM University Hospital, Munich, Germany

**Keywords:** Coronary calcification, Computed tomography, CAD screening, Agatston score, Non-invasive imaging, CAC

## Abstract

**Background:**

Coronary artery calcium (CAC) scoring is an established marker of atherosclerotic burden and cardiovascular risk. While the Agatston score is the clinical gold standard, alternative visual scoring methods—including the Visual Ordinal Score, Weston Score, and Vessel-specific extent-based score—are increasingly used, particularly in non-gated or opportunistic CT imaging. This study aimed to compare the diagnostic performance, inter-observer reliability, and correlation of different visual scoring methods against the Agatston score.

**Methods:**

A total of 299 cases were evaluated using ECG-gated CT scans. Each case was independently scored in a blinded fashion by two observers using three visual methods: (1) Visual Ordinal Score (VS), (2) Weston Score (WS) and (3) Vessel-specific extent-based score (VSES). A novel visual CAC score was derived by combining Weston and Vessel-specific extent-based scoring (= Weston Extent Score, WES). Cohen’s Kappa and Intraclass Correlation Coefficients (ICC) were used for inter-observer agreement. Classification performance was assessed against Agatston-based categories (No CAC, Mild, Moderate, Severe), including accuracy, precision, sensitivity, and specificity. Correlation analyses were conducted using Pearson and Spearman coefficients.

**Results:**

All scoring methods showed high correlation with the Agatston score (Spearman ρ > 0.87; p < 0.001). Visual scoring demonstrated the highest inter-observer agreement (Kappa = 0.94, ICC = 0.97), followed by Weston (Kappa = 0.90) and Vessel-Specific scores (Kappa = 0.77). Visual scoring also yielded the highest accuracy (Observer 1: 91.3 %, Observer 2: 90.0 %) The newly derived WES score achieved 80.9 % accuracy, with macro-averaged specificity of 93.8 % and improving diagnostic accuracy compared to WS and VSES.

**Discussion:**

Different visual scoring offers excellent reproducibility and diagnostic accuracy for CAC classification, with strong correlation to the Agatston score. The newly-derived WES score could be useful in providing a practical balance regarding volumetric information (CAC densitiy) and anatomical distribution of CAC. These findings support the implementation of structured visual CAC scoring in clinical and opportunistic CT settings.

## 1 Introduction

Coronary artery calcium (CAC) is a well-established biomarker of coronary atherosclerosis and plays a critical role in contemporary cardiovascular risk assessment. The presence and burden of CAC correlate strongly with future cardiovascular events and mortality, making it an important tool for refining risk prediction, particularly in asymptomatic individuals at intermediate cardiovascular risk. The Agatston score, derived from ECG-gated non-contrast computed tomography (CT), is the most validated and widely used method to quantify CAC. It is endorsed by major societies, including the American Heart Association (AHA), the American College of Cardiology (ACC), and the European Society of Cardiology (ESC) for guiding preventive therapy decisions in select patients aged 40 to 75 years [1], [2].

Despite its diagnostic accuracy and established prognostic value, the Agatston method has practical limitations. It requires ECG-gated acquisition, which is not typically available in general radiology settings, and relies on specialized post-processing software for automated plaque quantification. These requirements restrict its use to dedicated cardiac imaging centers and limit its routine application, especially in patients undergoing CT imaging for non-cardiac indications. At the same time, non-gated thoracic CT scans are frequently performed in clinical practice for a wide range of non-cardiac reasons, including lung cancer screening, oncologic staging, evaluation of interstitial lung disease, and preoperative planning. These scans often encompass the coronary arteries, providing an opportunity for “opportunistic” CAC detection. Several studies have demonstrated that incidentally noted CAC on non-gated CT is a strong predictor of cardiovascular outcomes [3], [4], [5]. However, CAC is frequently underreported in such settings, which represents a missed opportunity for early cardiovascular risk identification and prevention [6].

In response to these challenges, several visual CAC scoring methods have been proposed that can be applied without ECG gating or advanced software. These include the Visual Ordinal Score (VOS), the Weston Score (WS), and vessel-specific extent-based scores (VSES) [7], [8], [9], [10], [11], [12], [13]. Although each of these methods has shown utility in prior studies, direct comparisons of their diagnostic performance and reproducibility remain limited. Furthermore, combining the complementary strengths of different approaches—such as the anatomical specificity of VSES with the density assessment of WS—may improve visual scoring accuracy and robustness. Such hybrid scores could serve as practical tools for routine and opportunistic CAC classification, especially in non-gated imaging environments.

Therefore, the aim of this study was twofold: (1) to compare the diagnostic performance, correlation with Agatston categories, and inter-observer agreement of three commonly used visual scoring systems (VOS, WS, and VSES), and (2) to propose and evaluate a newly developed composite score—the Weston-Extent Score (WES)—which integrates both density and extent-based information. This approach seeks to identify a simple yet effective scoring strategy for broader implementation of CAC evaluation in both cardiac and non-cardiac CT settings.

## 2 Methods

### 2.1 Study population

This retrospective, stratified sub-study included 299 adult patients who underwent cardiac CT examinations for suspected coronary artery disease (CAD) at the TUM University Hospital German Heart Center, Germany, from October 2020 to September 2021. Patients were included if an Agatston score had been previously calculated as part of routine clinical evaluation. Exclusion criteria were the presence of significant motion artifacts, prior coronary artery bypass grafting (CABG), or missing image data. Patients with acute coronary syndrome, life-threatening conditions, unstable sinus rhythm during the examination, or a history of stent implantation or coronary bypass surgery were excluded from the analysis. Prior to the examination, a structured interview was conducted, collecting information on the patient’s age, height, weight, medical history, current symptoms, and medications. The study design was approved by the local ethics committee (Nr. 2023-628-S-SB).

### 2.2 CT image analysis

All cardiac CT examinations were performed using a 192-slice dual-source CT system (Siemens Medical Solutions, Erlangen, Germany). ECG-gated, non-contrast CT scans were performed prior to coronary CT angiography (CCTA) as part of the standard protocol to exclude CAD. For calcium score analysis, axial thin-slice images were reconstructed with a slice thickness of 3 mm and an increment of 1.5 mm, acquired at end-diastole. The Agatston score was calculated using commercially available software (Syngo.via VV80D, Siemens Healthineers, Erlangen, Germany) in accordance with standard guidelines. Based on the Agatston score, patients were stratified into four categories: no CAC (score = 0), mild CAC (1–99), moderate CAC (100–299), and severe CAC (≥300). Three visual coronary calcium scoring methods were independently applied by two readers with high expertise in cardiovascular CT imaging (Observer 1: senior radiologist; Observer 2: senior cardiologist).

### 2.3 Coronary calcium assessment

**Fig. 1:**
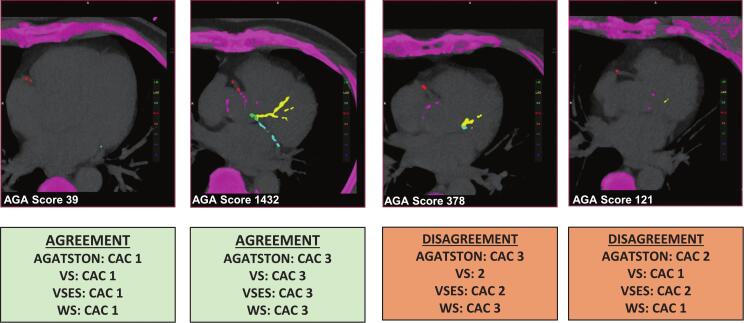
**Examples of agreement and disagreement between Agatston and visual CAC scoring methods.** Representative chest CT images illustrating concordant and discordant classifications between the Agatston score (AS), Visual Score (VS), Vessel-Specific Extent Score (VSES), and Weston Score (WS). Cases of agreement demonstrate consistent categorization across methods, while disagreement cases highlight borderline findings where visual scores may under- or overestimate CAC severity compared with AS.

The following scoring systems were used (for examples, see Fig. 1**)**. For details on the scoring systems, please see [14].

**Visual Ordinal Score:** A 4-point scale categorizing overall CAC as 0 = none, 1 = mild, 2 = moderate, and 3 = severe.

**Weston Score:** A semi-quantitative method scoring each major coronary artery (left main, LAD, LCx, RCA) on a 0–3 scale depending on calcification density; total score range 0–12.

**Vessel-Specific Score:** A segmental approach scoring each major vessel based on the extent of calcification (0–3); total score range 0–12.

**Weston-Extent Score:** A new composite metric was also calculated as the sum of the Weston and vessel-specific scores for each patient (range: 0–24),) with score 0 = no CAC, 1–5 = mild CAC, 6–8 = moderate CAC, and ≥9 = severe CAC.

### 2.4 Statistical analysis

Descriptive statistics were used to summarize baseline characteristics. Agreement between readers was assessed using Cohen’s Kappa for categorical scores and Intraclass Correlation Coefficient (ICC) for continuous scores. Diagnostic performance of each score was evaluated by calculating accuracy, precision, sensitivity, and macro-averaged specificity, using the Agatston score categories as the reference standard. Correlations between the Agatston score and each visual scoring method were assessed using Spearman’s rank correlation coefficient (ρ) and Pearson’s correlation coefficient (r).

Category thresholds for WES were determined during exploratory analyses. Using Agatston-based risk categories (0, 1–99, 100–299, ≥300) as the reference standard, multiple candidate cut-off schemes were evaluated. Ordinal agreement between WES categories and Agatston categories was quantified using quadratic-weighted Cohen’s κ, which accounts for chance agreement and penalizes distant misclassification. The cut-off values 0 = no CAC, 1–5 = mild CAC, 6–8 = moderate CAC, and >9 = severe CAC demonstrated excellent agreement with Agatston categories (κ = 0.91) and were therefore selected for subsequent analyses.

Owing to the highly skewed distribution of coronary calcium scores, Agatston values were transformed using the decadic logarithm (log10[1 + CAC]) prior to regression analyses. Linear regression models were applied to assess the relationship between log-transformed Agatston scores and each visual scoring method. For each model, we report the slope, corresponding 95 % confidence interval (CI), p-value, and coefficient of determination (R^2^). Scatterplots with regression lines were generated to illustrate relationships. All analyses were performed using SPSS (version 28.0). A p-value < 0.05 was considered statistically significant.

## 3 Results

### 3.1 Study Cohort

The study population consisted of 299 patients who underwent non-contrast ECG-gated CT imaging (Supplemental Table 1). For baseline data, please see Supplemental Table 2. Based on Agatston scoring, 50 patients (16.7 %) had no coronary artery calcium (CAC; Agatston score = 0), 100 patients (33.4 %) were categorized as having mild CAC (Agatston score 1–99) with a mean score of 24.7, 50 patients (16.7 %) had moderate CAC (Agatston score 100–299; mean score 187.1), and 99 patients (33.1 %) were classified as having severe CAC (Agatston score ≥ 300; mean score 635.9).

### 3.2 Inter-observer agreement

Inter-observer agreement was assessed for all three visual CAC scoring methods. The highest agreement was observed for the visual score, with a Cohen’s Kappa of 0.94 and an intraclass correlation coefficient (ICC) of 0.97, indicating excellent reliability (Table 1) The Weston score demonstrated similarly high reproducibility, with a Kappa of 0.90 and an ICC of 0.97 (Table 2). The vessel-specific score showed slightly lower agreement between observers, with a Kappa of 0.77 and an ICC of 0.83, corresponding to substantial to excellent agreement (Table 3).

**Table 1:** Diagnostic Performance and Correlation of the Visual Score Compared to Agatston Score.

**Metric**	**Observer 1**	**Observer 2**
**Accuracy (%)**	91.3	90.0
**Precision (%)**	91.7	90.7
**Sensitivity (%)**	91.3	90.0
**Specificity (macro, %)**	97.0	96.8
**Correct Classifications**	273 / 299	269 / 299
**Incorrect Classifications**	26	30
**Spearman’s ρ (p-value)**	0.96 (p < 0.001)	0.96 (p < 0.001)
**Pearson’s r (p-value)**	0.94 (p < 0.001)	0.94 (p < 0.001)
**Slope [95 % CI]**	0.99 [0.95–1.03]	1.00 [0.96–1.04]
**R^2^**	0.89	0.89
**Cohens Kappa**	0.94	
**Intraclass Correlation (ICC)**	0.97	

Summary of classification performance and correlation coefficients for the Visual CAC Score in comparison to the Agatston score. Shown are results for both observers. Specificity is macro-averaged across Agatston-defined CAC categories.

**Table 2:** Diagnostic Performance and Correlation of the Weston Score Compared to Agatston Score.

**Metric**	**Observer 1**	**Observer 2**
**Accuracy (%)**	75.3	75.3
**Precision (%)**	86.5	83.1
**Sensitivity (%)**	75.3	75.3
**Specificity (macro, %)**	92.5	92.4
**Correct Classifications**	225 / 299	225 / 299
**Incorrect Classifications**	74	74
**Spearman’s ρ (p-value)**	0.96 (p < 0.001)	0.95 (p < 0.001)
**Pearson’s r (p-value)**	0.88 (p < 0.001)	0.86 (p < 0.001)
**Slope [95 % CI]**	2.96 [2.80–3.13]	3.17 [2.99–3.34]
**R^2^**	0.81	0.81
**Cohens Kappa**	0.90	
**Intraclass Correlation (ICC)**	0.97	

Performance and correlation of the Weston CAC Score with the Agatston score for both observers. Specificity is macro-averaged across all CAC categories.

**Table 3:** Diagnostic Performance and Correlation of the Vessel-Specific Extend-Based Score Compared to Agatston Score.

**Metric**	**Observer 1**	**Observer 2**
**Accuracy (%)**	72.2	69.6
**Precision (%)**	62.7	58.0
**Sensitivity (%)**	72.2	69.6
**Specificity (macro, %)**	89.6	89.0
**Correct Classifications**	216 / 299	208 / 299
**Incorrect Classifications**	83	91
**Spearman’s ρ (p-value)**	0.90 (p < 0.001)	0.87 (p < 0.001)
**Pearson’s r (p-value)**	0.86 (p < 0.001)	0.82 (p < 0.001)
**Slope [95 % CI]**	1.37 [1.28–1.46]	1.70 [1.56–1.83]
**R^2^**	0.74	0.68
**Cohens Kappa**	0.77	
**Intraclass Correlation (ICC)**	0.83	

Performance and correlation metrics of the vessel-specific CAC Score against Agatston scoring for both readers. Specificity is macro-averaged across CAC classes.

### 3.3 Performance of the visual Ordinal score (VOS)

**Fig. 2:**
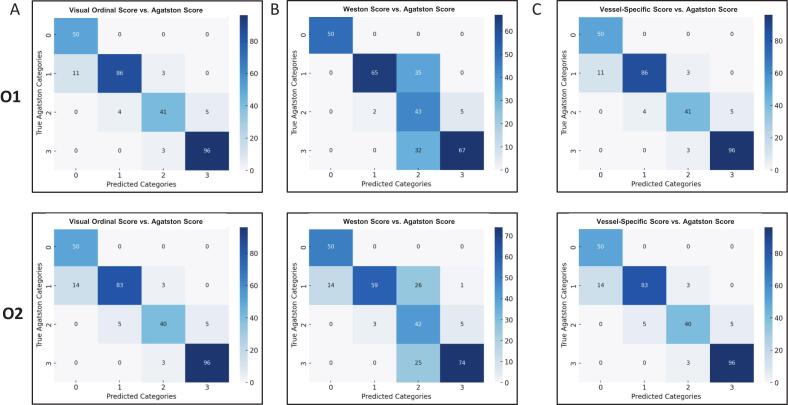
**Confusion matrices of visual scoring methods compared with the Agatston score.** Confusion matrices display the classification performance of (A) the Visual Ordinal Score (VOS), (B) the Weston Score (WS), and (C) the Vessel-Specific Extent Score (VSES). Upper rows represent observer 1 (O1) and lower rows observer 2 (O2). Each matrix compares visual categories against Agatston-defined CAC severity categories (0 = none, 1 = mild, 2 = moderate, 3 = severe). Correct classifications are shown along the diagonal, while off-diagonal cells indicate misclassification patterns.

**Fig. 3:**
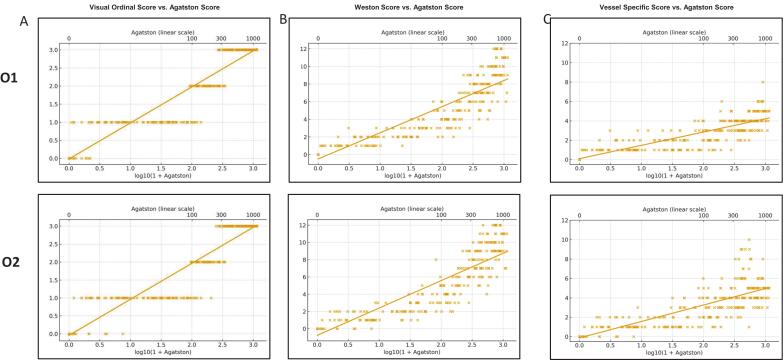
**Linear regression plots illustrating the association between log-transformed Agatston scores and conventional visual scoring methods. **Scatterplots with regression lines are shown for (A) Visual Ordinal Score (top: observer 1/O1; low: observer 2/O2) B) Weston Score and C) Vessel-specific Score. The x-axis represents log10(1 + Agatston), with a secondary axis (top) indicating corresponding untransformed Agatston values for clinical interpretability.

The visual ordinal score showed the best overall diagnostic performance among the scoring methods (Table 1). Classification accuracy was 91.3 % for observer 1 and 90.0 % for observer 2. Precision values were 91.7 % and 90.7 %, respectively. Sensitivity reached 91.3 % (Observer 1) and 90.0 % (Observer 2), and macro-averaged specificity was 97.0 % (Observer 1) and 96.8 % (Observer 2). A total of 273 cases were correctly classified by Observer 1 and 269 by Observer 2 with 26 and 30 misclassifications, respectively (Fig. 2A). Visual ordinal scores demonstrated a strong correlation with the Agatston score (Fig. 3A). Spearman’s rank correlation was ρ = 0.96 (p < 0.001) for both observers. Pearson’s correlation coefficients were r = 0.94 (p < 0.001) for both observers. Regression slopes were 0.99 (95 % CI [0.95–1.03], p < 0.001, R^2^ = 0.89) for observer 1 and 1.00 (95 % CI [0.96–1.04], p < 0.001, R^2^ = 0.89) for observer 2.

### 3.4 Performance of the Weston score (WS)

The Weston score achieved a classification accuracy of 75.3 % for both observers (Table 2). Precision was 86.5 % for Observer 1 and 83.1 % for observer 2. Sensitivity was 75.3 % in both cases, while macro-averaged specificity reached 92.5 % (observer 1) and 92.4 % (observer 2). Each observer correctly classified 225 of 299 cases, with 74 misclassifications (Fig. 2B). Correlations with the Agatston score were also strong (Fig. 3B). Spearman’s rank correlation was ρ = 0.96 (p < 0.001) for observer 1 and ρ = 0.95 (p < 0.001) for observer 2. The corresponding Pearson coefficients were r = 0.88 (p < 0.001) and r = 0.86 (p < 0.001), respectively. Regression slopes were 2.96 (95 % CI [2.80–3.13], p < 0.001, R^2^ = 0.81) for observer 1 and 3.17 (95 % CI [2.99–3.34], p < 0.001, R^2^ = 0.81) for observer 2.

### 3.5 Performance of the vessel-specific extent-based score (VSES)

The vessel-specific score yielded a classification accuracy of 72.2 % for observer 1 and 69.6 % for observer 2 (Table 3**).** Precision was calculated at 62.7 % (O1) and 58.0 % (O2), with sensitivity matching the respective accuracy. Macro-averaged specificity was 89.6 % for O1 and 89.0 % for O2. Correct classification was achieved in 216 cases by O1 and in 208 cases by O2, while 83 and 91 cases were misclassified, respectively (Fig. 2C). Correlation with the Agatston score was slightly lower than for the other scoring systems (Fig. 3C**)**. For observer 1, Spearman’s ρ was 0.90 (p < 0.001) and Pearson’s r was 0.79 (p < 0.001). For observer 2, the values were ρ = 0.87 (p < 0.001) and r = 0.70 (p < 0.001). Regression slopes were 1.37 (95 % CI [1.28–1.46], p < 0.001, R^2^ = 0.74) for observer 1 and 1.70 (95 % CI [1.56–1.83], p < 0.001, R^2^ = 0.68) for observer 2.

### 3.6 Performance of the newly-developed Weston-Extent score (WES)

**Fig. 4:**
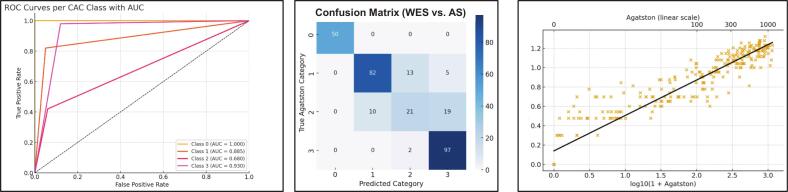
**Diagnostic performance of the newly developed WES score compared with the Agatston score.** Confusion matrix, classification and scatterplot results for the novel WES score (integration of Weston and Vessel-Specific scores) benchmarked against the Agatston score.

A combined WES score was derived by adding the Weston and Vessel-Specific Extend-Based scores (Table 4**)**. Using the cut-offs 0 = no CAC, 1–5 = mild CAC, 6–8 = moderate CAC, and ≥ 9 = severe CAC, the WES score reached a classification accuracy of 83.6 %. Precision was 82.8 %, and sensitivity matched accuracy at 83.6 %. Macro-averaged specificity was calculated at 94.2 %. Overall, 250 cases were correctly classified, while 49 were misclassified. Receiver operating characteristic (ROC) analysis showed high discriminative power for no CAC (AUC = 1.000) and severe CAC (AUC = 0.930), with slightly lower AUC values for mild (0.885) and moderate CAC (0.680, Fig. 4).

**Table 4:** Diagnostic Performance of the newly-developed Weston-Extent Score (WES) Compared to Agatston Score.

**Metric**	
**Accuracy (%)**	83.6
**Precision (%)**	82.8
**Sensitivity (%)**	83.6
**Specificity (macro, %)**	94.2
**Correct Classifications**	250 / 299
**Incorrect Classifications**	49
**Spearman’s ρ**	0.95 (p < 0.001)
**Pearson’s r**	0.95 (p < 0.001)
**Slope [95 % CI]**	0.37 [0.35–0.38]
**R^2^**	0.91

Diagnostic performance of the combined WES score, derived by summing Weston and vessel-specific scores. Classification was based on predefined thresholds: 0 = no CAC, 1–5 = mild, 6–8 = moderate, ≥9 = severe.

## 4 Discussion

This study provides a comprehensive head-to-head comparison of three visual coronary artery calcium (CAC) scoring systems—Visual Ordinal Score (VOS), Weston Score (WS), and Vessel-Specific Extent Score (VSES)—against the reference standard Agatston score, using ECG-gated CT scans. In addition, we proposed and validated a novel composite metric, the Weston-Extent Score (WES), which integrates both plaque density and anatomical distribution. Our findings highlight the clinical and diagnostic utility of visual CAC scoring, offer comparative insights into their strengths and limitations, and support the incorporation of pragmatic CAC evaluation into routine imaging workflows.

**Key findings of our study were as follows:**

1. All visual scoring methods correlated strongly with the Agatston score (Spearman’s ρ ≥ 0.87), underscoring their value as surrogate tools for CAC classification in both clinical and opportunistic imaging scenarios.
2. The Visual Ordinal Score (VOS) achieved the highest diagnostic accuracy and inter-observer agreement among the tested methods, with a classification accuracy of 91.3 % and excellent reproducibility (κ = 0.94, ICC = 0.97).
3. The Weston-Extent Score (WES), a new composite metric, demonstrated balanced performance, combining the strengths of WS and VSES. It achieved an accuracy of 83.6 % and a macro-averaged specificity of 94.2 % with an R^2^ = 0.91, outperforming its individual components.

These results are consistent with prior studies showing that visual CAC scoring can serve as a reliable and reproducible alternative to automated Agatston quantification, particularly in contexts where gated acquisition and post-processing tools are not available [7], [13]. The VOS performed particularly well, confirming previous reports that this simple, intuitive method correlates strongly with Agatston categories and has high diagnostic utility, especially for categorical classification (e.g., presence vs. absence of CAC) [8]. However, its subjectivity remains a concern, especially in scans with borderline or ambiguous calcification, and requires training and expertise for optimal inter-reader consistency.

The WS has been used extensively in non-cardiac imaging cohorts, including studies in chronic obstructive pulmonary disease (COPD), idiopathic pulmonary fibrosis (IPF), and emergency department settings [15], [9], [10], [11]. While it demonstrated strong correlation with Agatston scores in our study, its lower classification accuracy (∼75 %) reflects a potential limitation: it does not capture the anatomical extent of disease, which may be particularly relevant in patients with diffuse, low-density calcification. Moreover, its semi-quantitative nature, while appealing in terms of speed, may sacrifice granularity and underestimate disease severity in certain anatomical distributions.

The VSES, by contrast, incorporates anatomical extent but excludes density information. This method showed the lowest overall performance in terms of accuracy and inter-observer agreement. Previous studies have similarly investigated the extent-based visual scores [12], [13], [16] However, while capturing spatial information this approach might misclassify patients with localized yet dense calcification, or those with subtle diffuse disease.

Our proposed WES sought to address these limitations by combining WS and VSES. Notably, this score maintained strong correlation with the Agatston standard (ρ = 0.95) and achieved a high overall classification accuracy of 83.6 %. Importantly, the observed performance exceeded that of the individual component scores (accuracy of 75.3 % for WS and 72.2 % for VSES), supporting the added diagnostic value of the composite metric in visual coronary calcium assessment. By combining the anatomical granularity of the VSES with the simplicity and established validity of the WS, the WES capitalized on the complementary strengths of both approaches. Receiver operating characteristic (ROC) analysis showed excellent discrimination for both CAC = 0 (AUC = 1.00) and severe CAC (AUC = 0.93), with slightly lower performance in intermediate categories. This pattern reflects the diagnostic strengths of the WES in “rule-in” and “rule-out” settings and could support its use in threshold-based clinical decision-making, such as identification of patients eligible for statin therapy.

### 4.1 Clinical implications

The clinical relevance of our findings is underscored by the growing recognition of CAC as a valuable tool beyond dedicated cardiac imaging. In our study, visual CAC scoring was intentionally evaluated under controlled conditions (ECG-gated CT scan) to establish a methodological benchmark, serving as a prerequisite for subsequent validation in opportunistic, non-gated CT environments. As non-gated chest CTs become increasingly common, particularly in oncology and pulmonary medicine, the opportunity for incidental detection of CAC grows. Unfortunately, underreporting of incidental CAC remains prevalent, with studies suggesting that such findings are frequently omitted from structured reports, even when visually apparent [6], [17]. Implementing standardized visual scoring systems, such as VOS or WES, could promote more consistent recognition and reporting of CAC in these settings. Benefits over the conventional Agatston Score could be summarized as follows:

1. Simplicity and Speed: Visual scoring can be performed more quickly without the need for complex software, allowing for immediate risk assessment during routine scans.
2. Resource Efficiency: Visual CAC scoring requires less specialized training and equipment, making it suitable for use in diverse healthcare settings, including resource-limited environments.
3. Broader Accessibility: Enables broader implementation in clinical practice where dedicated CAC scoring software or expertise may not be available.
4. Potential for Integration with Routine Imaging: Can be incorporated into existing non-contrast CT scans performed for other indications, optimizing patient evaluation without additional procedures

From a public health and preventive cardiology perspective, early identification of asymptomatic individuals with significant CAC may facilitate initiation of risk-reducing interventions, such as lifestyle counseling or statin therapy. Visual scoring tools, by virtue of their accessibility and low cost, could support population-level screening strategies, particularly in resource-limited settings or in patients undergoing non-cardiac CT for other indications [18], [19]. Moreover, integration of visual scores into structured reporting systems or radiology AI platforms could further streamline their adoption [20], [21], [22], [23]. Our study also has implications for future research. Few studies to date have evaluated composite visual scores that combine plaque density and distribution. Further studies are needed to assess its prognostic value in terms of cardiovascular outcomes and its performance in non-gated CT cohorts. Additionally, validation in diverse clinical populations and relevant subgroups would broaden generalizability.

### 4.2 Limitations

Several limitations warrant mention: first, although the primary clinical motivation of visual coronary artery calcium (CAC) scoring lies in its applicability to opportunistic, non–ECG-gated chest CT examinations, all analyses in the present study were performed on ECG-gated CT scans acquired under optimized cardiac imaging conditions. Consequently, our results may overestimate diagnostic performance and inter-observer agreement compared with non-gated CT, where cardiac motion, lower temporal resolution, and variable image quality may adversely affect visual CAC assessment. While prior studies have demonstrated that visual CAC scoring retains diagnostic and prognostic value in non-gated settings, performance metrics may be attenuated in routine clinical practice [4], [24], [25]. Therefore, extrapolation of our findings to opportunistic non-gated CT should be performed with caution, and further validation in non-gated chest CT cohorts is warranted. In addition, visual CAC scoring was performed by two highly experienced cardiovascular imaging readers (a senior radiologist and a senior cardiologist), which may have contributed to the high inter-observer agreement observed in this study. Reproducibility and diagnostic performance may be lower when visual scoring is applied by readers with less experience in cardiovascular CT or in general radiology practice. Therefore, our agreement metrics likely represent best-case performance and may not be fully generalizable to all clinical settings. Future studies should assess inter-observer reliability across different levels of reader training and expertise. Furthermore, this study was designed as an imaging-based methodological comparison and did not include long-term clinical outcome data. As a result, the prognostic implications of the visual scoring methods could not be assessed. Although baseline demographic and clinical characteristics were collected and are provided in the Supplement, the present analysis did not include predefined subgroup analyses according to age, sex, or cardiovascular risk profile. Consequently, the generalizability of our findings across different patient subgroups remains limited and should be addressed in future outcome-oriented and population-based studies.

In conclusion, our study demonstrates that visual CAC scoring methods provide reliable and reproducible alternatives to Agatston scoring, particularly in contexts where ECG-gated CT and automated tools are unavailable. The Visual Ordinal Score offers excellent performance for categorical risk classification, while the newly developed Weston-Extent Score provides a balanced, composite approach that integrates density and extent information. Both methods hold promise for broader use in clinical and opportunistic CT imaging, potentially expanding access to cardiovascular risk stratification across healthcare settings.

## Grant support

None.

## CRediT authorship contribution statement

**Philipp Nicol:** Writing – original draft, Supervision, Investigation, Formal analysis, Data curation, Conceptualization. **Rafael Adolf:** Writing – review & editing, Investigation, Formal analysis, Data curation. **Salvatore Cassese:** Writing – review & editing, Supervision, Resources, Methodology. **Adnan Kastrati:** Writing – review & editing, Supervision, Resources, Formal analysis. **Michael Joner:** Writing – review & editing, Supervision, Resources, Methodology, Formal analysis. **Heribert Schunkert:** Writing – review & editing, Supervision, Resources. **Martin Hadamitzky:** Writing – review & editing, Methodology, Data curation, Conceptualization. **Leif-Christopher Engel:** Writing – original draft, Supervision, Investigation, Formal analysis, Data curation, Conceptualization.

## Declaration of competing interest

The authors declare that they have no known competing financial interests or personal relationships that could have appeared to influence the work reported in this paper.
