# Supplementary material for: Visual coronary calcium scoring to support opportunistic CAD screening: comparative evaluation of three established systems and introduction of a novel scoring system
Source: Int J Cardiol Heart Vasc. 2026 Jan 19;62:101875. doi: 10.1016/j.ijcha.2026.101875 (PMC12856988; doi:10.1016/j.ijcha.2026.101875)
Supplement: Supplementary Data 1 [file mmc1.docx]

**Supplemental Table 1. Study Cohort Characteristics by Agatston Score Category**

| **Agatston CAC Category** | **No. of Patients** | **Mean Agatston Score** |
| --- | --- | --- |
| 0 (No CAC) | 50 | 0 |
| 1 (Mild: 1–99) | 100 | 24.7 |
| 2 (Moderate: 100–299) | 50 | 187.1 |
| 3 (Severe: ≥300) | 99 | 635.9 |

**Legend:**
*Distribution of 299 patients according to Agatston-based CAC severity groups, including the average Agatston score per group.*

**Supplemental Table 2. Baseline Characteristics of the Study Population with CAC >0 (n = 299).**

| **Characteristic** | **Value** |
| --- | --- |
| **Demographics** |  |
| Age (years) | 65 [58–75] |
| Male sex, n (%) | 191 (76.7) |
| Body Mass Index (kg/m²) | 24.5 [21.6–28.4] |
|  |  |
| **Cardiovascular Risk Factors** |  |
| Diabetes mellitus, n (%) | 27 (13.1) |
| Hypercholesterolemia, n (%) | 148 (71.5) |
| Hypertension, n (%) | 126 (60.0) |
| Smoking (current or former), n (%) | 71 (34.0) |
| Family history of MI, n (%) | 69 (34.0) |
|  |  |
| **Symptoms (NYHA Class)** |  |
| NYHA 0, n (%) | 22 (10.9) |
| NYHA I, n (%) | 130 (64.7) |
| NYHA II, n (%) | 40 (19.9) |
| NYHA III, n (%) | 9 (4.5) |
|  |  |
| **Angina Pectoris Type** |  |
| No angina, n (%) | 142 (70.0) |
| Atypical angina, n (%) | 55 (27.1) |
| Non-anginal chest pain, n (%) | 4 (2.0) |
| Stable angina, n (%) | 2 (1.0) |
|  |  |
| **Laboratory Parameters** |  |
| Total cholesterol (mg/dL) | 196 [159–239] |
| LDL cholesterol (mg/dL) | 119 [87.5–157] |
| HDL cholesterol (mg/dL) | 57 [47–68.3] |
| Triglycerides (mg/dL) | 112 [80.5–158] |
| Framingham risk score (%) | 10.8 [6.0–16.9] |
| **Indication for Cardiac CT** |  |
| Arrhythmia, n (%) | 33 (13.3) |
| Preoperative evaluation, n (%) | 19 (7.6) |
| Dyspnea, n (%) | 18 (7.2) |
| Chest pain, n (%) | 55 (22.1) |
| Risk profile assessment, n (%) | 93 (37.3) |
| Ischemia, n (%) | 23 (9.2) |
| Triple-rule-out, n (%) | 4 (1.6) |
| Other indications, n (%) | 3 (1.2) |
| Unknown/unspecified, n (%) | 1 (0.4) |

*Values are presented as median [interquartile range] or number (percentage), as appropriate.
NYHA = New York Heart Association functional class; MI = myocardial infarction; LDL = low-density lipoprotein; HDL = high-density lipoprotein; CT = computed tomography.*
